# Concurrent cisplatin, continuous infusion fluorouracil and radiotherapy followed by tailored consolidation treatment in non metastatic anal squamous cell carcinoma

**DOI:** 10.1186/1471-2407-11-55

**Published:** 2011-02-03

**Authors:** Maria G Zampino, Elena Magni, Maria C Leonardi, Luigi Santoro, Elena Petazzi, Cristiana Fodor, Giuseppe Petralia, Cristina Trovato, Franco Nolè, Roberto Orecchia

**Affiliations:** 1 Medical Care Unit, Department of Medicine, European Institute of Oncology, via Ripamonti 435, Milan 20141, Italy; 2 Radiotherapy Division, European Institute of Oncology, via Ripamonti 435, Milan 20141, Italy; 3 Epidemiology and Biostatistics Division, European Institute of Oncology, via Ripamonti 435, Milan 20141, Italy; 4 Radiology Division, European Institute of Oncology, via Ripamonti 435, Milan 20141 Italy; 5 Endoscopy Division, European Institute of Oncology, via Ripamonti 435, Milan 20141, Italy; 6 University of Milan, via Ramusio 1, Milan 20141, Italy

## Abstract

**Background:**

To evaluate efficacy and feasibility of chemo-radiotherapy in patients with non-metastatic anal squamous-cell-cancer.

**Methods:**

TNM staged anal squamous-cell cancer patients were treated with pelvic radiotherapy concomitant to continuous infusion fluorouracil plus cisplatin for at least 2 cycles. In T3-T4 or any T - N+ tumours or in "slow-responder" cases, 1-2 chemotherapy courses were subsequently administered. Tumour assessment was performed at baseline and 6-8 weeks after radiotherapy to evaluate response.

**Results:**

29 patients were enrolled: 4 males, 25 females; median age 57 years; baseline T1/T2/T3/T4 2/12/7/8; N involvement 17. Median dose pelvic radiotherapy was 59.4 Gy (range: 54-74). In 5 patients 2 chemotherapy courses, in 12 patients three and in 12 patients four courses were performed. At first evaluation, 27 CR (93.1%; 95% CI: 78% - 98%) and 2 SD were observed. Main grade (G) 3 toxic events were neutropenia (8%), diarrhoea (8%) and dermatitis (62%). Most frequent late events G3-G4 occurred in 14 patients: proctitis (5), dermatitis (4), bladder dysfunctions (2), sexual dysfunctions (9), lower extremity venous thromboses (2), dysuria (1), stenosis (1) and tenesmus (1). Five patients reported G1 leucopoenia. The rate of colostomy was 14%. After a median follow up of 42 months (range: 4-81), 20 patients are still alive without relapse and 3 died due to PD. The estimated 7-year DFS was 83.4% (C.I.: 68.3%-98.5%) and the estimated 7-year OS was 85.7% (C.I.: 70% - 100%). The 1-year and the estimated 7-year colostomy-free survivals were 85.9% (C.I.: 73.1% - 98.7%).

**Conclusions:**

Concurrent cisplatin plus fluorouracil and radiotherapy is associated with favourable local control rates and acute toxicity. Future investigations will be directed towards research into molecular biomarkers related to disease progression and resistance to chemo-radiotherapy and to the evaluation of new cytotoxic agents or targeted drugs, such as anti-epidermal growth factor receptor, concomitant to RT and to determining the role of intensity-modulated radiotherapy.

## Background

Anal carcinoma is an uncommon disease that represents approximately 1% of all gastrointestinal malignancies with increasing incidence over the past 25 years. Risk factors include HPV-infection, immune depression-suppression, and smoking. Loco-regional progression often occurs while metastatic potential is present in 15% of patients [1]. Localized stage and a radio-chemotherapeutic sensitive tumour represent favourable conditions suitable to curative non-surgical treatment. The conventional approach with external-beam radiation therapy (RT) with concomitant fluorouracil (FU) and mitomycin (M) has induced successful disease control in two thirds of patients [2]. An anal lesion size greater than 5 cm and regional node involvement are two clinical features that have resulted in a reduction of the cure rate (local control 50-60%) and worst outcome; a more aggressive approach is needed in this setting of patients [3]. With the aim of increasing objective response and reducing haematologic toxicity related to M, cisplatin (C) was investigated in clinical trials, due to its property as radiation sensitizer and its therapeutic potential in squamocellular histology, and the interesting results also seen in anal cancer. But the optimal duration and the best chemotherapy-combination containing C, have not been yet determined. Our mono-institutional phase II trial aimed to evaluate FU as continuous infusion (c.i.) plus C in patients affected by non-metastatic anal squamous cell cancer (SCC) with primary tumours larger than 2 cm. The number of cycles was tailored on baseline patients characteristics and tumour response at the end of RT: one or two additional courses after RT were planned in high risk patients with T >5 cm and/or node-positive disease at baseline or in cases of persistence of primary lesion with regression <50% (slow responders) at the end of RT. The rationale of such a strategy was justified by early use of radiotherapy combined with radiosensitive agents and also in order to evaluate the role of systemic therapy if administered after the end of radiotherapy.

Primary goals were complete remission (CR) and disease-free survival (DFS) and secondary aims were colostomy rate and overall survival (OS).

## Methods

### Patients and inclusion criteria

We undertook a prospective study with chemo-radiotherapy, in patients affected by T2-T4 anal carcinoma or any T with locoregional positive nodes. Eligibility criteria included: histologically proven anal squamous cell carcinoma, age ≥ 18 years, ECOG performance status ≤ 2, adequate haematological, liver and renal function (granulocyte count >1500/mm^3^, platelet count >100000/mm^3^, haemoglobin >10 g/dl, serum creatinine <1,25 Upper Limit of Normal (ULN), serum bilirubin <1,25 ULN, GOT or GPT <2 ULN), absence of metastases, no prior systemic chemotherapy (CT) or RT to the pelvis, life expectancy ≥ 3 months. Patients affected by uncompensated cardiopathy or recent acute myocardial infarction, peripheral neuropathy, uncontrolled infection, inflammatory bowel disease or history of another tumour, were excluded. Initial clinical work-up was performed with a digital examination of the anus, rectal wall and recto-vaginal septum, ano-rectal endoscopy and tumor biopsy, chest-abdomino-pelvic CT scan, routine blood tests. The clinical T-stage and nodal involvement were determined by anorectal endoscopic ultrasound (EUS) or by pelvic MRI. Patients with obstructive lesion underwent temporary colostomy before commencement of the study. In all patients a central vein catheter was positioned for chemotherapy delivery. All patients signed a clinical trial informed consent form that was notified to the European Institute of Oncology Ethical Committee.

### Treatment protocol

Radical radiotherapy was delivered by using a high energy linear accelerator (≥15 MV or mixed energy). Customized blocking for conformal techniques and immobilization system were used in all cases. The gross tumor volume (GTV), clinical target volume (CTV) and planning target volume (PTV) and organs at risk (OARS) were contoured using an Eclipse treatment planning system (Varian Medical Systems, Palo Alto Ca, USA). GTV included the primary tumor and any involved lymph nodes. Two CTVs were considered: a CTV1 which included the GTV, anal canal and a uniform margin of 1 cm; and a CTV2 which included regional lymph nodes at risk such as the external iliac, internal iliac, perirectal, obturator, presacral and the inguinal lymph nodes. PTV 1 and PTV2 were generated by adding a uniform 1.5 cm margin around the CTV1 and CTV2.

PTV2 is treated up to 36 Gy, 1.8 Gy per fraction, in 22 fractions with anterior and posterior (AP/PA) opposing fields with the superior border of the field at L5/S1 interspace and in nodal positive patients up to 45 Gy in 25 fractions of 1.8 Gy with the superior field lowered to the bottom of the sacroiliac joints using either AP/PA fields or a box-technique.

The planning goals were to deliver to the PTV1, via external beam radiotherapy, a dose ranging from a minimum of 41.4 Gy in case of T1-T2 tumors to a maximum of 59.4 in 1.8 Gy per fraction, by using multiple-field technique. Brachytherapy (BRT) was allowed to be administered to the primary tumor either in T1-T2 patients in order to spare OARs after PTV2 phase completion or in T3 patients with a slow tumor response after the maximum prescription external dose of 59.4 Gy. The large variability in dose prescription and in administration modality reflected the different physicians' attitudes and any eventual treatment adjustment in accordance with patient compliance.

Concurrent chemotherapy containing FU 200 mg/m2 i.v. daily as 24 hours continuous infusion administered via a portable pump through central vein port-a-cath and at least 2 cycles of C 80 mg/m2 days 1;21 iv were planned in an outpatient setting. One course was defined as one administration of C plus 3 weeks of c.i. FU.

In patients with T3-T4 or nodal involvement at baseline work-up or in "slow-responder" cases (persistence of local disease with regression <50%) evaluated by at least two physicians at the end of RT, 2 chemotherapy courses were considered. After 6 and within 8 weeks after concomitant chemoradiotherapy, patients underwent tumour reassessment with clinical and instrumental evaluation with ano-rectal EUS and/or pelvic MRI. Tumoral biopses were planned only in case of suspicious progressive local disease. Subsequent tumour evaluation in non-progressive cases was repeated every 3 months for the first 2 years and every 6 months thereafter. Chest and abdominal CT scan was performed every 6 months for 2 years and every 12 months thereafter.

### Statistical design

Response was evaluated according to Word Health Organization (WHO) criteria [4]. Acute toxicity was assessed using the National Cancer Institute Common Toxicity Criteria (NCI-CTC) scale, version 2.0, combined to Radiation Therapy Oncology Group (RTOG) scale; late toxicity using SOMA-LENT scale. Treatment was delayed for toxicity G >2 except for nausea or vomiting until G1 or complete recovery. If chemotherapy was delayed more than 3 weeks, the patient was withdrawn. This phase II trial was planned using Simon's Statistical Single Stage Design. For sample size calculation power was assumed as 80% and type I error (alpha) as 5%. A CR rate of 70% or smaller was considered unacceptable, whereas a response rate of 90% or greater was considered sufficient to justify further investigations. The single-stage design required the enrolment of 28 patients; in cases of 24 responses or more the regimen would be considered worthy of further investigations. Descriptive statistics were provided either by median and range for continuous variables or frequency for categorical variables. Response rates and toxicities were summarised by the 95% confidence interval; interval estimation was provided by the Wilson formulae [5]. Disease-free survival was estimated from the end of chemo-radiotherapy (or date of colostomy when done) to relapse, death or last known follow-up. Overall survival was estimated from the start-time of chemo-radiotherapy to death or last known follow-up. The Kaplan-Meier approach was used for the probability estimation. All analyses were carried out using SAS statistical software (SAS Institute, Cary, NC).

## Results

### Patient characteristics

From June 1999 to June 2008, 29 patients were considered eligible for the study. Main patient characteristics were described in Table 1: stage II 9 patients (8 T2/N0 and 1 T3/N0), stage III A 14 patients (1 T1/N1, 4 T2/N1, 6 T3/N1, 3 T4/N0) stage IIIB 6 patients (1 T1/N3, 1 T4/N1, 1 T4/N2, 3 T4/N3). All patients completed chemo-radiotherapy and were considered evaluable for response. Median duration of CT-RT was 11 weeks (8-17), with a median length of external RT of 49 days (range 29-84). The pelvic lymph nodes (PTV2) received a median dose of 41.4 Gy (range 36.0-46.8) through external RT, while the primary tumor and involved nodes received a median dose of 59.4 Gy (range 54.4-74 Gy), either using external RT alone or combined with BRT. With regard to BRT, a median dose of 15 Gy (range 4-30 Gy) on primary tumor was given to 12 patients after a median interval from the end of RT of 3.2 weeks (range 0.1-5.6). Nine of them were treated by High Dose Rate/Pulsed Dose Rate technique by using a cylindrical applicator while in 3 patients, interstitial implant of BRT was performed under anaesthesia with a perineal template. Total median RT dose in patients with Stage T1 was 59.4 Gy (range 59.4-59.4); T2 62.8 (range 54.4-74); T3 59.4 (56.4-69); T4 59.4 (59,4-70.4). Stage T2 received a larger total dose compared to more advanced tumours: this can be explained by the more frequent use of BRT boost which increases total dose.

**Table 1:** Patients' Characteristics

Variables	Number
Median age (yrs)	57 (range 40-75)

Gender	
Males	4 (14%)
Females	25 (86%)

Grading	
G1	1 (3%)
G2	13 (45%)
G3	8 (28%)
Unknown	7 (24.1%)

Performance status (ECOG)	
0	20 (71%)
1	7 (25%)
2	1 (4%)

Concomitant Pathologies	15 (52%)

Clinical staging	
T1N1	1 (3%)
T1N3	1 (3%)
T2N0	8 (28%)
T2N1	4 (14%)
T3N0	1 (3%)
T3N1	6 (21%)
T4N0	3 (10%)
T4N1	1 (3%)
T4N2	1 (3%)
T4N3	3 (10%)

Tumor location	
Anal canal	20 (71%)
Anal canal + anal margin	8 (28%)
Anal margin	1 (3%)

### Treatment Response

**Figure 1:**
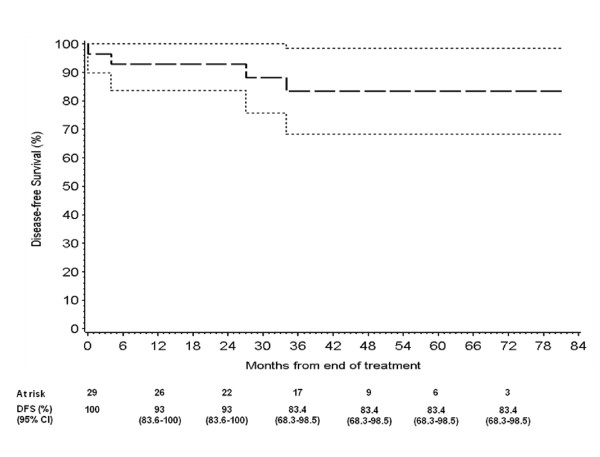
**3- 5- and 7-years Disease-Free Survival**.

**Figure 2:**
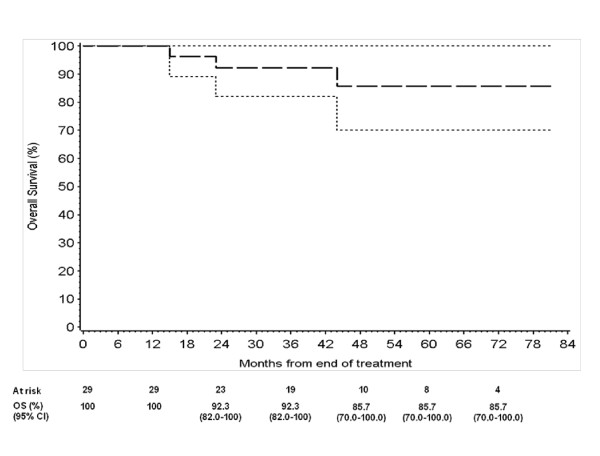
**3- 5- and 7-years Overall Survival**.

Two months after the end of chemoradiotherapy, in 27 out of 29 patients (93.1%; 95% CI: 78% - 98%) CR was documented, while in two patients a stable disease (SD) was reported. One of them presented CR at subsequent tumor assessment, 3 months later and the other progressed after few months. Two patients were diverted pre-treatment due to local disease extension and one of them underwent successful reversal after nine months from the end of RT; in the remaining one, persistent anal sphincter incontinence was observed. During treatment in one case colostomy was considered due to sub-occlusion determined by treatment-related congestive tumour response. After chemoradiotherapy two patients underwent abdominal perineal resection for local disease persistence, but in one case no residual tumour was documented in the surgical specimen. The rate of colostomy was 14%. The 1-year and the estimated 7-year colostomy-free survival were 85.9% (C.I. 73.1% - 98.7%). Over time three patients with CR, exhibited systemic relapse at 4 and 34 months. One patient died and the other is still alive at 19 months since evidence of relapse. After a median follow up of 42 months (range: 4-81), 25 patients are still alive without relapse, 1 patient is alive with relapse and 3 patients died due to PD. The estimated 3, 5 and 7-year DFS were 83.4% (C.I. 68.3%-98.5%) and the estimated 3-year OS was 92.3 (range 82%-100%) and 5 and 7-year OS were 85.7% (C.I. 70% - 100%) (Figure 1; Figure 2).

DFS for patients who underwent a colostomy without evidence of disease, was censored at the date of colostomy.

### Delivered Treatment and Toxicity

During treatment 14 patients discontinued chemoradiotherapy due to RT side effects, which lasted with as median 9 days (range 1-39) (Table 2). No chemotherapy delay more than 3 weeks was observed. At the 2^nd ^month, 11 patients reported toxicities mostly related to RT; two events were classified as G3-G4 (1 proctitis and 1 rectal bleeding). Concerning chemotherapy a total of 98 cycles were administered: FU was given in 97, while C was given in 94. (Table 3) FU and C were given together in 93 courses and in 62 without delays/modifications; in 4 cases and 1, FU and C were given as single agent, respectively. All patients received at least 2 cycles of C: 5 patients (17.2%) received two cycles, 12 patients (41.4%) three and 12 patients (41.4%) four cycles. The rate of patients who performed 4 cycles was 21.4% among T1/T2; 57.1% among T3 and 62.5% among T4 stage. In 70 cycles out of 94 (74.5%) CT full dose-intensity was administered.

**Table 2:** RT on-treatment toxicity

Variables	Number
	29

No of patients with at least 1 toxicity	29 (100%)
Maximum grade toxicity	
*G2*	*16*
*G3*	*12*
*G4*	*1*

No of side effects	95
*G1*	*17*
*G2*	*63*
*G3*	*14*
*G4*	*1*

No. of patients with gastrointestinal toxicity	26 (89.7%)
No. of GI side effects	49
*Abdominal pain*	5 (2)*
*Diarrhoea*	14 (1)*
*Mucorrhea*	*6*
*Nausea/vomiting*	*6*
*Proctitis*	*6*
*Rectal bleeding*	*2*
*Rectal pain*	*4*
*Constipation*	*2*
*Tenesmus*	*4*

No. of patients with genito-urinary toxicity	10 (37.9%)
No. of GU side effects	11
*Dysuria*	*11*

No. of patients with other toxicities	26 (37.9%)
No. of Other toxicities	35
*Dermatitis*	25 (9)*
*Mucositis*	10 (3)*

* In parentheses the number of G3-G4 events

**Table 3:** Chemotherapy characteristics and on-treatment toxicities

Variables	Number
Total number of cycles	98

Fluorouracil	97^

Cisplatin	94*
Patients receiving	
2 cycles	5 (17.2%)
3 cycles	12 (41.4%)
4 cycles	12 (41.4%)

Cisplatin modification (total # of cycles = 94)	
Full dose-intensity	70 (74.5%)
Delay/modification	24

Cisplatin and/or Fluorouracil modification (total # of cycles over the first 2 cycles = 58)	
Full dose-intensity	49 (70.7%)
Delay/modification	9
Reasons	
Haematological toxicities	**2**
*Neutropenia (G3)*	*1*
*Leucopoenia (G1)*	*1*
Non Haematological toxicities	**5**
*Diarrhoea (G2)*	*1*
*Nausea (G2 and G3)*	*2*
*Nausea and Diarrhoea (G2)*	*1*
*Mucositis (G2)*	*1*
Haematological and non-haematological toxicities	**1**
*Diarrhoea (G2) and Leucopoenia (G2)*	*1*
Other toxicities	**1**
*Hypoacusia (G2) *	*1*

^ 1 patient received C only

* 5 patients received Fluorouracil only

Focusing on the first two mandatory cycles, 20 patients out of 29 received a full-intensity dose while in 9 patients the 2^nd ^cycle was delayed or modified due to 1 G3 neutropenia and 2 G1-2 leucopoenia, 6 G2-G3 non-haematological toxicities. At 6 months after the end of RT, 6 patients did not exhibit any treatment related toxicity. Most frequent late events G3-G4 occurred in 14 patients: proctitis (5), dermatitis (4) bladder dysfunctions (2), sexual dysfunctions (9), low extremities venous thromboses (2), dysuria (1), stenosis (1) and tenesmus (1). Five patients reported G1 leucopoenia (Table 4).

**Table 4:** Late toxicity

	Number of Events		
**Variables**	**Grade 1-2**	**Grade 3**	**Grade 4**

Dysuria/Incontinence	2/4	2/-	-/1

Abdominal Pain	4	-	-

Rectal Pain	4	1	-

Bleeding	10	-	-

Ulceration	-	3	1

Anorectal Stenosis	-	1	-

Female Sexual Dysfunction	15	10	8

Male Sexual Dysfunction	-	1	-

Deep Venous Thrombosis	-	2	-

Bone Fracture	-	1	-

Leucopoenia	5	-	-

## Discussion

Concurrent chemoradiotherapy is the standard treatment for localized anal cancer, while surgical resection has been reserved as salvage strategy only for persistent disease and for local relapse. However, it often fails to control spread of the disease. With M and FU containing CT-RT 60-90% local control rates and 60-70% 5-year survival rates were reported, with infrequent need for colostomy [1,6,7].

Mitomycin added to FU shows improved results in terms of local control and time to colostomy in 3 randomized trials compared to RT alone and RT plus FU as single agent [2,8,9]. Unfortunately M can cause severe, life-threatening haematological adverse events (18%), lung toxicity, and haemolytic-uremic syndrome.

Alternative regimens containing C, cytotoxic agent with considerable interest as a radiation sensitizer, have been investigated in phase II trials, even if no definite role of this agent is well determined by now (Table 5) [6,10-13].

**Table 5:** Cisplatin-containing Phase II Trials

Author	Trial, Total number patients and Stage	Regimen	Response	Toxicity ≥ G3
Martenson, 1996	Phase II: 19 pts T1-4/N0-3/M0	2 cycles FU 1000 mg/m2 × 4 days, CDDP 75 mg/m2 concomitant to RT 59.4 Gy	15 CR (79%); 4 PR (21%) Colostomy rate: na DFS: na OS: na	G3-4: haematological 50% diarrhoea 20% skin 20% G5: 1 infection

Doci, 1996	Phase II: 35 pts T1-T3/N1-3/M0	2-3 cycles FU 750 mg/m2 × 4 days, CDDP 100 mg/m2 concurrent RT 36-38 Gy	33 CR (94%) 2 PR (6%) Median follow-up 37 mos. colostomy free 86% OS 94%	G3: Diarrhoea 2-3% skin 2-3%

Gerard, 1998	Phase II: 95 pts T1-4/N0-3/M0	1 cycle FU 1000 mg/m2 × 4 days, CDDP 25 mg/m2/d × 4 days concomitant to RT followed by a boost with 192 Ir implant.	85 CR (89%) 7 PR (8%) Median follow-up 64 mos. 5y colostomy free 71% 5yOS 84%	G3: haematological 3% TVP 1%

Peiffert, 2001	Phase II: 80 pts T1-4/N0-3/M0	2 neoadjuvant and 2 cycles FU 800 mg/m2 × 4 days, CDDP 80 mg/m2 concomitant to RT 45 Gy	70 CR 70 (87%) 4 PR (5%) At 3 year: colostomy-free 73%; OS 86%	G3-G4: angina pectoris 2% mucositis 16% diarrhoea 16% skin 32% G5: 1 TEP

Cho, 2008	Phase II: 31 pts T1-4/N0-3/M0	2 cycles FU 750-1000 mg/m2 days 1-5, CDDP 75-100 mg/m2 concomitant to RT 45 Gy →2 consolidation cycles FU +CDDP	31 CR (90.3%) 4 PR (9.7%) Median follow-up 72 mths; DFS 82.9% OS 84.7%	G3-G4: skin 55% neutropenia 33% infection 3.2% fatigue 4.8%

Zampino, Current work	Phase II: 29 pts T1-4/N0-3/M0	2-4 cycles FU 200 mg/m2 i.c CDDP 75 mg/m2 day1/21 concomitant to RT 45-74 Gy	Median follow-up 7 yrs; CR 93% DFS 85% OS 86%	G3-G4: mucositis 10% diarrhoea 3% skin 31% Hematological 3%

In our phase II trial the role of early C-containing chemotherapy and concurrent radiotherapy followed by the same regimen administered for one or two courses, was investigated in order not to delay RT, considered as an irreplaceable therapeutic procedure in the treatment of anal SCC and in order to evaluate additional therapy in high risk cases. In this population of patients, 93.1%; (95% CI: 78% - 98%) of CR was reported at 2 months with an estimated DFS of 83.4% (68.3%-98.5%) and estimated 7-years OS of 85.7% (70% - 100%). These data confirm the efficacy of C-containing schemes as previously reported in phase II trials. The results of our study might be related to better radiosensitizer action of FU administered as continuous infusion and to tailored consolidation CT courses delivered in cases of high risk baseline stage and 'slow response' after concomitant chemo-radiotherapy. As for colostomy on 27 evaluable patients (two patients had colostomy before treatment), 3 patients (11%) derived during or after chemo-radiotherapy. Concerning acute toxicity, we observed perineal mucosa and skin side effects of grade 3-4 in about 40% of patients, similar to previously reported literature data (Table 4). Grade 3-4 diarrhoea was observed in less than 5% of patients, with a lower incidence compared to that reported by older series (30-44%) [9,14,15]. The modern use of 3D conformal RT with optimized protection of normal tissues, the technique of shrinking fields, and devices for sparing the small bowel may clearly contribute to reduce these side effects [15,16]. The literature-reported rate of late toxicity, varies from 4% to 50% depending on follow-up length, radiation treatment parameters such as RT technique and fraction site, and chemotherapeutic agents combination [17]. In our series 23 patients had at least one G2, while 15 at least one G3-G4 late toxicity. The highest toxicity rate was the rectal one (16 patients), with G3 reported in 5 patients. Among other G3-G4 toxicity, we observed 10 cases of sexual dysfunction (9 F and 1 M), 5 anal ulceration, 4 dermatitis, 3 urinary events, 1 vulvo-vaginitis, 1 bone fracture. Toxic late events reported in our series seem to be similar to previous reported literature data, except for sexual discomfort which was often not described [6,10-12]. Intracavitary or interstitial RT was used to deliver a boost to the tumor bed, while sparing surrounding normal structures.

The incidence of G≥2 late side effects was higher in patients receiving a boost with external RT than those receiving it by BRT: among the 12 BRT patients, 5 (41.7%) suffered from side effects higher than G2 as maximum toxicity compared to 10 out of 17 patients (58.8%) in external RT group. In particular, all the BRT boost patients experienced a lower bladder toxicity (8.3% vs. 41.2%, p 0.05) and a slight higher rectal toxicity (58.3 vs. 52.9, p 0.77) compared external RT boost patients. In the BRT boost group, no relationship between rectal maximum toxicity and total dose was found, G ≥2 toxicity was observed in all 3 patients treated with interstitial modality and in 3 out of 9 patients treated with intracavitary modality.

Recently the impact of C as an attractive drug was investigated in phase III trials with the aim to evaluate neoadjuvant treatment before concomitant chemoradiotherapy [7,18-20]. In the Intergroup RTOG 98-11 phase III trial the comparison was evaluated between C plus FU induction treatment followed by the same chemotherapy and concurrent radiation and M plus FU and concurrent radiation: C-based therapy failed to improve disease-free-survival compared with M-control arm, and resulted in a significantly worse colostomy rate (19% vs 10%); severe hematological toxicity was confirmed to be worse with the M-containing arm [7].

It must be emphasized that the RTOG 98-11 trial was not a pure comparison of concurrent chemoradiation with C plus FU versus M plus FU but, rather, was a comparison of one strategy versus another. The assumption that the strategy of induction chemotherapy had been able to reduce the bulk of local-regional anal SCC was not supported by the results documented in randomized study.

Also in the ACCORD 03 trial neoadjuvant role of C-FU was investigated[19] Early data on quality of life has already been published [20]. Combination of C and M was also evaluated in a phase III trial and compared to conventional FU-M concomitant to RT: local control was reported in 89% vs 74%, respectively with acceptable toxicity [21].

At the moment these results do not support the use of C-based neoadjuvant chemotherapy followed by chemoradiotherapy in place of M in combination with FU and radiotherapy in the treatment of anal canal carcinoma.

## Conclusions

The promising results obtained in present study even though observed in a limited number of patients, seemed to be suitable for future investigation with the aim to confirm the possible use of early administered C-containing treatment with radiotherapy and to evaluate the advantage of sequential chemotherapy after chemoradiotherapy in high risk patients with anal SCC.

Further research into molecular biology related to disease progression and resistance to chemoradiotherapy may provide a better understanding of different pattern of anal carcinoma and may also support the clinical use of biologic agents. Future investigations will be directed towards the evaluation of new cytotoxic agents such as taxanes or targeted drugs such as anti-epidermal growth factor receptor, concomitant to RT and to determining the role of intensity-modulated radiotherapy [22,23].

## Competing interests

The authors declare that they have no competing interests.

## Authors' contributions

MZ, EM, CL made substantial contributions to the conception and design, acquisition analysis and interpretation of data; were involved in drafting the manuscript and gave final approval of the version to be published. EP, CT, GP made substantial contributions to the conception and design, acquisition analysis and interpretation of data. CF made substantial contributions to acquisition analysis and interpretation of data. LS participated in the design of the study and performed the statistical analysis. FN, RO were involved in revising the manuscript for important intellectual content. All authors read and approved the final manuscript.

## Pre-publication history

The pre-publication history for this paper can be accessed here:

http://www.biomedcentral.com/1471-2407/11/55/prepub
